# Equine Herpesvirus Type 1 ORF76 Encoding US9 as a Neurovirulence Factor in the Mouse Infection Model

**DOI:** 10.3390/pathogens13100865

**Published:** 2024-10-02

**Authors:** Mohamed Nayel, Samy Kasem, Noriko Fukushi, Nagwan El-Habashi, Ahmed Elsify, Akram Salama, Hany Hassan, Tokuma Yanai, Kenji Ohya, Hideto Fukushi

**Affiliations:** 1 Department of Applied Veterinary Sciences, United Graduate School of Veterinary Sciences, Gifu University, 1-1 Yanagido, Gifu 501-1193, Japan; mohamed.aboalez@vet.usc.edu.eg (M.N.); samykasem2@gmail.com (S.K.); fukushi-noriko@g.ecc.u-tokyo.ac.jp (N.F.); kohya@nihs.go.jp (K.O.); 2 Department of Animal Medicine and Infectious Diseases, Faculty of Veterinary Medicine, University of Sadat City, Sadat City 32897, Egypt; ahmed.elsaify@vet.usc.edu.eg (A.E.); akram.salama@vet.usc.edu.eg (A.S.); hanyhassan1959@gmail.com (H.H.); 3 Department of Virology, Faculty of Veterinary Medicine, Kafr Elsheikh University, Kafr Elsheikh 33516, Egypt; 4 Department of Pathology, Faculty of Veterinary Medicine, Kafr Elsheikh University, Kafr Elsheikh 33516, Egypt; nagwan_hab@yahoo.com; 5 Department of Veterinary Pathology, Faculty of Applied Biological Science, Gifu University, 1-1 Yanagido, Gifu 501-1193, Japan; tokumayanai@gmail.com

**Keywords:** EHV-1, neurovirulence, US9, ORF76

## Abstract

Equine herpesvirus type 1 (EHV-1) causes rhinopneumonitis, abortion, and neurological outbreaks (equine herpesvirus myeloencephalopathy, EHM) in horses. EHV-1 also causes lethal encephalitis in small laboratory animals such as mice and hamsters experimentally. EHV-1 ORF76 is a homolog of HSV-1 US9, which is a herpesvirus kinase. Starting with an EHV-1 bacterial artificial chromosome clone of neuropathogenic strain Ab4p (pAb4p BAC), we constructed an ORF76 deletion mutant (Ab4p∆ORF76) by replacing ORF76 with the rpsLneo gene. Deletion of ORF76 had no influence on replication, cell-to-cell spread in cultured cells, or replication in primary neuronal cells. In Western blots of EHV-1-infected cell lysates, an EHV-1 US9-specific polyclonal antibody detected multiple bands ranging from 35 to 42 kDa. In a CBA/N1 mouse infection model following intranasal inoculation, the parent and Ab4p∆ORF76 revertant caused the same histopathology in the brain and olfactory bulbs. The parent, Ab4p∆ORF76, and revertant mutant replicated similarly in the olfactory mucosa, although Ab4p∆ORF76 was not transported to the olfactory bulbs and was unable to infect the CNS. These results indicated that ORF76 (US9) plays an essential role in the anterograde spread of EHV-1.

## 1. Introduction

Equine herpesvirus type 1 (EHV-1) (*Varicellovirus equidalpha1*) (ICTV, https://ictv.global/taxonomy, accessed on 23 September 2024) causes rhinopneumonitis, abortion, and neurological outbreaks (equine herpesvirus myeloencephalopathy, EHM), which occur independently or synchronously [1]. Saxegaard (1966) first reported the isolation of EHV-1 from a case with neurological signs [2]. Cases of EHM have been reported with increasing frequency and severity in Europe and the USA [3,4,5,6]. EHM causes disastrous losses in the equine industry [5,7]. The neurological signs ranged from mild ataxia to paraplegia in horses and may be caused by ischemic degeneration resulting from vasculitis, hemorrhage, and thrombosis [6,8]. A point mutation in the EHV-1 DNA polymerase gene (ORF30) was highly associated with EHM [9]. The finding that field isolates that are both associated and not associated with EHV-1 neurological disease and the results of experimental infection and molecular characterization studies support the hypothesis that EHM is strongly correlated with a single nucleotide polymorphism of EHV-1 DNA polymerase gene [10,11,12,13].

EHV-1 has been isolated from fatal encephalitis outbreaks in non-equine species, including gazelle, giraffes, and polar bears [14,15,16,17,18]. Thus, EHV-1 infection is a threat to the zoo and captive animals as well as horses. Viral factors causing fatal encephalitis in non-natural hosts should be clarified for future prevention and therapy of herpesvirus infections.

Ab4p is a plaque clone of the neurovirulent strain Ab4 that was isolated from EHM [19,20] and has been shown to cause nervous manifestations in experimentally infected horses, hamsters, and mice [19,21,22]. We have used hamster and mouse infection models to study the neurovirulence of EHV-1 [23,24]. EHV-1 might enter the brain through the nasal mucosa and olfactory bulbs [25]. An EHV-1 infectious BAC (pAb4pBAC) was established from the Ab4p [23]. Using this BAC system, UL24 encoded by ORF37 of EHV-1 was reported to be a neurovirulence factor in a mouse infection model [23]. The Ab4p BAC and Ab4p attB, which is a virus after removal of the BAC sequence from Ab4p BAC, represent important tools for studying EHV-1 virulence and neuropathogenesis.

EHV-1 ORF76 encodes a tegument protein US9 [20]. EHV-1 US9 homologs are highly conserved in herpesviruses including herpes simplex virus-1 (HSV-1) [26,27,28], varicella-zoster virus (VZV) [29], pseudorabies virus (PRV) [30], simian herpesvirus B [31], bovine herpesvirus 1 (BHV-1) [32], feline herpesvirus 1 (FHV-1) [33], canine herpesvirus [34], and herpes simplex virus -2 (HSV-2) [35]. A critical component of the life cycle of alphaherpesviruses is anterograde spread, which is the spread of the virus from the neuron cell body to the axon terminus. US9 has been found to be essential for the anterograde spread of PRV [36], BHV-1 [37], and bovine herpesvirus 5 (BHV-5) [38] as well as a determinant of neurovirulence and neuroinvasiveness of these viruses. However, it is unclear whether EHV-1 US9 has the same functions.

In this study, we used Ab4p BAC to construct US9 deletion mutant and revertant viruses to study the functions of EHV-1 US9. Our results suggest that US9 is necessary for the anterograde spread of EHV-1 from the olfactory epithelium to the olfactory bulbs.

## 2. Materials and Methods

### 2.1. Viruses and Cells

EHV-1 Ab4p BAC and Ab4p attB were used [23]. Fetal equine kidney (FEK) cells were cultivated with Dulbecco’s modified Eagle’s medium supplemented with 5% fetal bovine serum (FBS). Madin–Darby bovine kidney (MDBK) and Rabbit kidney 13 (RK13) cells were cultivated with MEM alpha supplemented with 5% FBS. 

### 2.2. Construction of Ab4pΔORF76 (US9 Deletion) and Revertant BACs

To construct Ab4pΔORF76 (US9 deletion), we used a RED/ET recombination system (counter-selection BAC modification system, Gene Bridges GmbH, Heidelberg, Germany) as described previously [23,39,40]. The primers used are shown in Table 1. The rpsLneo cassette (rpsLneo gene) was amplified by PCR using primers 1 and 2 and rpsLneo template DNA. The PCR product was used to replace ORF76 in pAb4p BAC, resulting in a recombinant BAC termed pAb4pΔORF76 BAC. For revertant Ab4pΔORF76R construction, the rpsLneo gene in the pAb4pΔORF76 BAC was further replaced by ORF76 PCR product amplified using primers 3 and 4, resulting in recombinant BAC termed pAb4pΔORF76R BAC. The constructs were sequenced to confirm the mutation and reversion. Restriction enzyme BamHI digestion patterns of each BAC DNA were examined to confirm the absence of extra changes in the viral genomes.

### 2.3. Recovery of Infectious Ab4p attB, Ab4pΔORF76, and Ab4pΔORF76R Viruses from BAC DNA

BAC DNA was extracted from *Escherichia coli* harboring pAb4p BAC, pAb4pΔORF76 BAC, and pAb4pΔORF76R BAC one by one using the Nucleo Bond BAC 100 kit (MACHEREY-NAGEL, Allentown, PA, USA). To recover Ab4p attB, Ab4pΔORF76, and Ab4pΔORF76R viruses without BAC sequence, BAC DNAs were treated with Gateway LR Clonase enzyme mix (Invitrogen, Life Technologies, Waltham, MA, USA) according to the manufacturer’s instructions. RK13 cells at 70–80% confluence in a 24-well plate were transfected with DNA using lipofectamine 2000 (Invitrogen, Tokyo, Japan) according to the manufacturer’s manual. After 5 days, the supernatant was collected to inoculate MDBK cells. The MDBK cells were overlaid by Eagle’s MEM (EMEM) (Nissui, Tokyo, Japan) containing 1.5% carboxymethylcellulose after 60 min of adsorption and incubated for 4–5 days at 37 °C. The desired virus plaques were identified and selected under fluorescent microscopy using green fluorescent protein (GFP) as a marker. Three rounds of plaque purification purified the recovered viruses. 

### 2.4. Time Course of Viral Growth 

The time course of viral growth was determined as described previously [41]. MDBK, RK13, and FHK monolayer cells in 24-well plates were inoculated with the indicated viruses at a multiplicity of infection (MOI) of 0.1. After 1.5 h of adsorption, cells were washed three times with EMEM and incubated at 37 °C in a 5% CO_2_ atmosphere in 0.5 mL/well of EMEM with 5% fetal calf serum. At the indicated times, cells were scraped together with culture fluids and centrifuged. The supernatants were used as the extracellular fluid samples. Sedimented cells were washed twice with EMEM, resuspended in 0.5 mL of EMEM, frozen and thawed three times, and centrifuged. The obtained supernatants were used as the intracellular fluid samples. Extracellular and intracellular fluid samples were titrated for viral infectivity by plaque assay as previously described [23]. 

### 2.5. Virus Growth Kinetics in Mouse Neurons

Growth kinetics of the viruses were measured in CX (M) mouse neurons (Sumitomo Bakelite, Tokyo, Japan). Neurons were cultured in 24-well plates coated with poly-L-lysine in a neuron culture medium (Sumitomo Bakelite, Tokyo, Japan) and infected with the various viruses at 1 MOI. At the indicated times, the supernatant and cells were separately harvested, and the titers of examined viruses were calculated using plaque assay as described previously [13].

### 2.6. Analysis of Transcription Kinetics by Real-Time RT-PCR

The transcription activity of ORF76 was evaluated by infecting MDBK cells with Ab4p attB, Ab4pΔORF76, and Ab4pΔORF76R at 1 MOI. Infected cells were harvested at 0, 2, 4, and 8 h post-infection. Total RNA was isolated using a Nucleospin RNA kit (MACHEREY-NAGEL, Allentown, PA, USA), and 1.5 µg of RNA was heated at 95 °C for 5 min for denaturation, combined with reverse transcriptase master mix (TOYOBO, Osaka, Japan). The mixture was incubated at 30 °C for 10 min, 42 °C for 40 min, and then stopped by heating at 99 °C for 5 min. The real-time PCR was carried out in a Thermal Cycler Dice Real Time System (TaKaRa, Kusatsu, Japan) using 12.5 µL of SYBR Premix Ex Taq (TaKaRa, Kusatsu, Japan), 10 µM of ORFs 76, 75, and 67 primers (Table 1), and 10 ng of cDNA. Relative quantities were measured with the ΔΔCt method [42]. 

### 2.7. Animal Experiments

Briefly, one hundred (four-week-old) specific-pathogen-free (SPF) male CBA/N1 mice (Japan SLC Corporation, Shizuoka, Japan) were equally divided into four groups: control, Ab4p attB, Ab4pΔORF76, and Ab4p ΔORF76R. Each group was inoculated with 1 × 10^5^ plaque forming unit (pfu) per head of the corresponding virus by the intranasal route. The body weight and behavior of the mice were observed from 3 days before inoculation until the end of the experiment. For virus isolation and DNA detection, two mice from each group were euthanized every day from 1 to 10 day post-infection (dpi). The brain, olfactory bulbs, and lungs were used for virological assay.

### 2.8. Preparation of Tissues for Virus Titration

Tissues from euthanized animals were homogenized in EMEM at 10% (*w*/*v*) and centrifuged at 3000 rpm for 10 min. The supernatant was 10-fold serially diluted in EMEM. A 24-well plate with a confluent MDBK monolayer was inoculated with 0.1 mL supernatant per well. Plaque assay was used for virus titration. The detection limit in the organ homogenates was 1 × 10^2^ pfu per gram. 

### 2.9. Preparation of Tissues for Virus DNA Detection by PCR

DNA was extracted from the organs of infected mice with a SepaGene kit (Sanko Junyaku, Tokyo, Japan). Viral DNA was detected by PCR with primers 3 and 4 for ORF76 and the rpsLneo gene inserted (Table 1). PCR amplification was performed in 50 µl volumes containing DNA (100 ng), 200 µM of each dNTP, 0.2 µM of each dNTP, 0.2 µM of each primer, 5 µL 10× Ex Taq Buffer and 1.25 U TaKaRa *Ex Taq*™ DNA Polymerase (5 units/µL) (TaKaRa, Kusatsu, Japan). The PCR conditions were as follows: 5 min at 94 °C (initial denaturation), 30 cycles of 5 s at 98 °C, 30 s at 68 °C and 90 s at 72 °C, and finally 7 min at 72 °C (final extension). The PCR product was separated on 0.9% agarose gel and stained with ethidium bromide. The expected sizes of the PCR products were 782 bp for ORF76 (Ab4p attB and Ab4p ∆ORF76R) and 1420 bp for rpsLneo inserted ORF76 (Ab4p ∆ORF76).

### 2.10. Histopathology and Immunohistochemistry

Tissues were collected, fixed in 10% buffered formalin, dehydrated, and embedded in paraffin wax by routine methods, sectioned at 5 μm, stained with hematoxylin and eosin (HE), and examined by light microscopy. Paraffin wax sections were immunolabeled with EHV-1 rabbit antiserum by the avidin–biotin complex (ABC) immunoperoxidase method with ABC kits (Vector Laboratories, Burlingame, CA, USA) as described previously [43]. The primary antibody was EHV-1 antiserum (1:1000, Veterinary Microbiology Laboratory at Gifu University), followed by the application of a secondary antibody (biotinylated anti-rabbit IgG, DAKO Cytomation, Carpinteria, CA, USA). The liquid DAB Substrate Chromogen System (DAKO Cytomation, Carpinteria, CA, USA) was used as chromogen and hematoxylin as a counterstain. Tissue sections from EHV-1-infected mice and sera from a non-immunized rabbit and mice were used as controls.

### 2.11. Immunofluorescence

An immunofluorescence assay was performed on paraffin wax sections as described previously [44]. The primary antibody was polyclonal US9 guinea pig serum (1:500, prepared in this study), followed by the application of a secondary antibody (FITC-labeled anti-guinea pig IgG, Sigma Aldrich, St. Louis, MO, USA). Fluorescent image analysis was examined using the Keyence Biozero system (Keyence, Tokyo, Japan).

### 2.12. Production of Anti-EHV-1 US9-Specific Polyclonal Antibody 

ORF76 (US9) was amplified using primers 5 and 6 (Table 1), which introduced an EcoRI site directly upstream of the start codon and a NotI site directly downstream of the US9 stop codon, respectively. The amplified PCR fragment was cloned into the EcoRI and NotI sites of pGEX-6P-1, generating pGST-US9 plasmid. Expression of GST-US9 fusion protein was induced by adding isopropyl-β-D-1-thiogalactopyranoside (IPTG, Takara, Shiga, Japan) to a culture of *E. coli* BL21 transformed with the pGST-US9 plasmid. The fusion protein was purified using glutathione-Sepharose 4B beads (GE Healthcare UK Ltd., Buckinghamshire, UK). The purified US9 was made into an emulsion by adding an equal volume of TiterMax^®^ Gold adjuvant (Funakoshi, Tokyo, Japan) and used to immunize two guinea pigs (Japan SLC Corporation, Shizuoka, Japan). Serum was collected before and after four subcutaneous applications of 100 µg protein at 1-week intervals.

### 2.13. Western Blotting

For Western blot analyses, RK13 cells were infected with viruses at a multiplicity of 5 pfu/cell and incubated at 37 °C for 1–18 h. Infected cells were harvested and pelleted by centrifugation at 14,000 rpm for 1 min in an Eppendorf centrifuge. Pellets were washed twice with phosphate-buffered saline (PBS), resuspended in 100 µL of PBS, mixed with the same volume of sample buffer, and heated at 95 °C for 5 min. Then, the samples were separated by SDS-PAGE and electrotransferred to nitrocellulose membranes (Millipore, Bedford, MA, USA). The blots were blocked with 5 % low-fat milk in Tris-buffered saline (TBS-T; 10 mM Tris/HCl, pH 8.0, 150 mM NaCl, 0.25% Tween 20) and incubated for 1 h with guinea pigs antisera against the US9 gene products at dilutions of 1:1000 in TBS-T. Bound antibody was detected with peroxidase-conjugated anti-guinea pig antibodies (Bethyl, Montgomery, TX, USA) and visualized by chemiluminescence (Amersham, Tokyo, Japan) and recorded on X-ray films.

### 2.14. Statistical Analysis

Data were analyzed using one-way ANOVA with Dunn’s multiple-comparisons test, and significance was set at *p* < 0.05 using RStudio 2024.04.2 software.

## 3. Results

### 3.1. Construction of ORF76 (US9) Deletion Mutant and Its Revertant Virus

**Figure 1:**
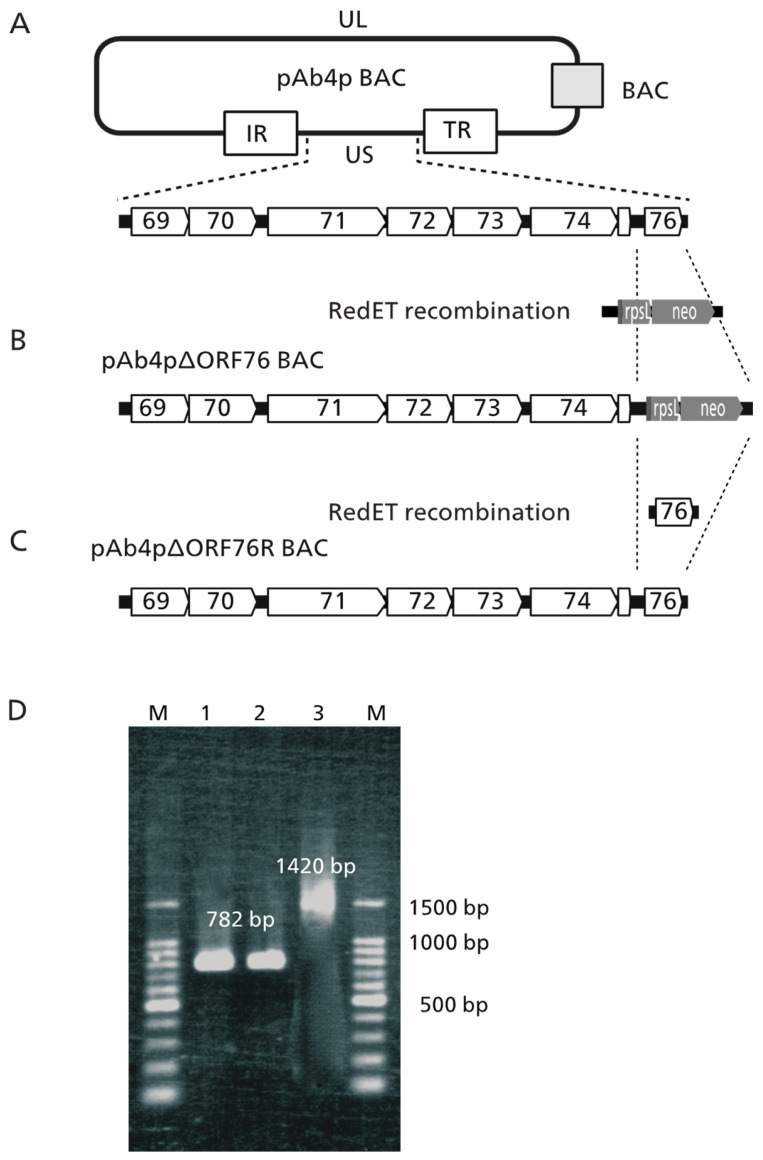
Construction and identification of EHV-1 ORF76 deletion and revertant mutant BAC. (**A**) The BAC plasmid, pAb4p BAC, including whole EHV-1 Ab4p genome maintained in E. coli DH10ß is represented as a round rectangle with a unique long (UL), an internal repeat (IR), a unique short (US), and a terminal repeat (TR) region. A grey rectangle represents a BAC vector sequence. Open arrows with numbers 69 to 76 are open reading frames of EHV-1. (**B**) Using RedET recombination system, ORF76 of pAb4p BAC was replaced with a rpsLneo cassette (pAb4p∆ORF76 BAC). (**C**) Using RedET recombination system, the rpsLneo of pAb4p∆ORF76 was repaired with ORF76 (pAb4p∆ORF76R BAC). (**D**) PCR identification of the generated recombinant viruses. Lane 1: Ab4p attB ORF76 (782 bp); Lane 2: Ab4pΔORF76R ORF76 (782 bp); Lane 3: Ab4pΔORF76 (rpsLneo gene 1420 bp); M: 100 bp ladder DNA molecular weight marker (100 bp) (TaKaRa, Kusatsu, Japan).

The ORF76 (US9) deletion mutant was constructed using pAb4p BAC with Red mutagenesis molecular recombination. The resulting ORF76 (US9) deletion mutant and revertant BAC plasmids were designated pAb4pΔORF76 (Figure 1B) and pAb4p∆ORF76R BAC (Figure 1C). The correct replacement and genotypes of the generated viruses were confirmed by PCR and sequencing. The 782 bp product, which indicated the presence of ORF76, was detected in cells infected with Ab4p attB and Ab4pΔORF76R viruses by PCR (Figure 1D, Lanes 1 and 2). A PCR product of 1420 bp was detected in cells infected with ORF76 deletion mutant Ab4pΔORF76 due to the presence of rpsLneo cassette instead of ORF76 (Figure 1D, Lane 3). The absence of extra changes in the viral genome was confirmed by BamHI digestion patterns of each BAC DNA.

### 3.2. Growth of ORF76 (US9) Deletion Mutant in Cultured Cells and Mouse Neurons

**Figure 2:**
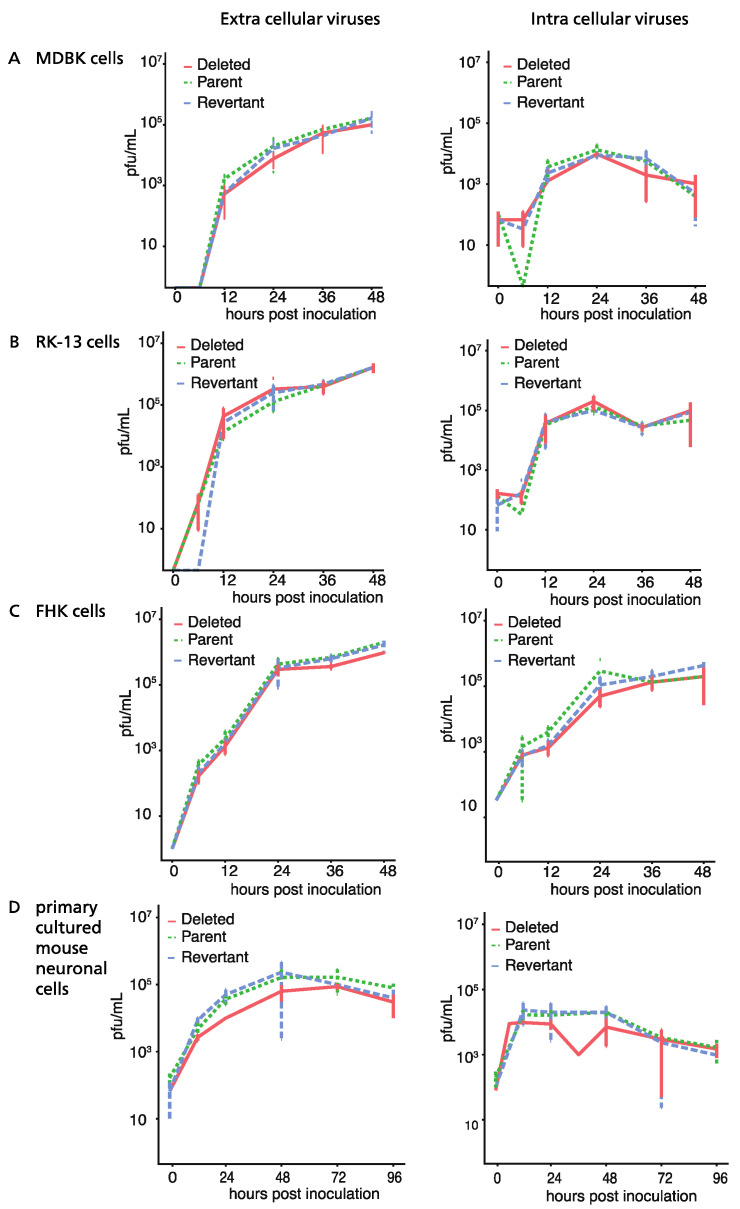
Mutant virus growth kinetics. The growth properties were evaluated by comparing the in vitro growth curves of Ab4p attB (Parent), Ab4pΔORF76R (Repvertant), and Ab4pΔORF76 (Deleted) in MDBK cells (**A**), RK-13 cells (**B**), FHK cells (**C**), and primary cultured mouse neuronal cells (**D**). The extracellular and intracellular viral titers of each examined virus were determined by plaque assay. The growth curves of the viruses showed the same patterns. Cells were infected at an MOI of 0.1, except primary cultured mouse neuronal cells, which were infected at an MOI of 1. Data were analyzed using one-way ANOVA with Dunn’s multiple-comparisons test. Error bars are standard errors.

Plaque sizes of the deletion mutant Ab4pΔORF76, the revertant Ab4p∆ORF76R, and the neuropathogenic strain Ab4p attB in cultured cells were similar. The infectious progeny yield and time course of Ab4p∆ORF76 were almost the same as those of Ab4p attB and Ab4p∆ORF76R in MDBK cells (Figure 2A), RK13 cells (Figure 2B) and FHK cells (Figure 2C). In addition, the growth kinetics of Ab4p∆ORF76 in primary cultured mouse neurons were similar to those of Ab4pΔORF76R and Ab4p attB (Figure 2D). These results show that ORF76 (US9) is not essential for infectious virus production in cultured cells or mouse neurons.

### 3.3. Effect of ORF76 Deletion on Transcription Activities of Other Genes 

**Figure 3:**
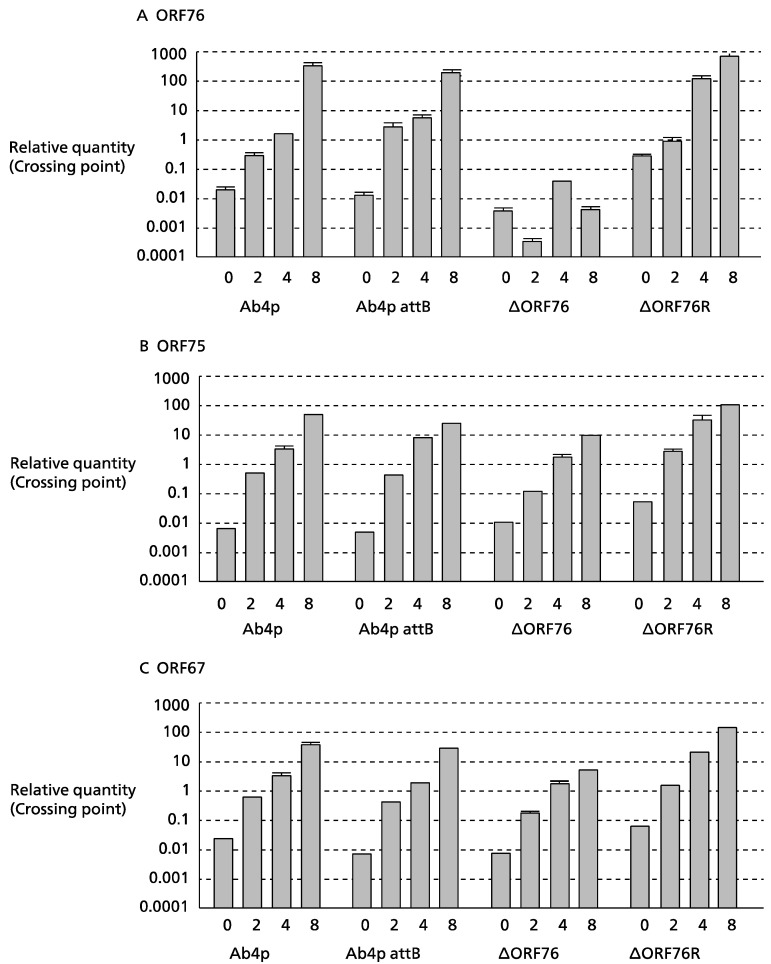
Transcription activity kinetics of ORF76 (**A**), ORF75 (**B**), and ORF67 (**C**) by real-time RT-PCR. RNA was extracted from infected MDBK cells harvested at 0, 2, 4, and 8 h post-infection (hpi). Relative quantity was evaluated by the crossing point method using the β-actin gene as 1 (control).

The expression levels of the β-actin gene (a control) in MDBK cells were the same for all viruses. No ORF76 transcripts were detected in cells infected with the Ab4p∆ORF76 deletion mutant (Figure 3C). ORF76 deletion had no effect on the transcription levels of two neighboring ORFs, ORF75 (Figure 3A) and ORF67 (Figure 3B), or a distant ORF (ORF30). 

### 3.4. Identification and Initial Characterization of EHV-1 US9 Protein

**Figure 4:**
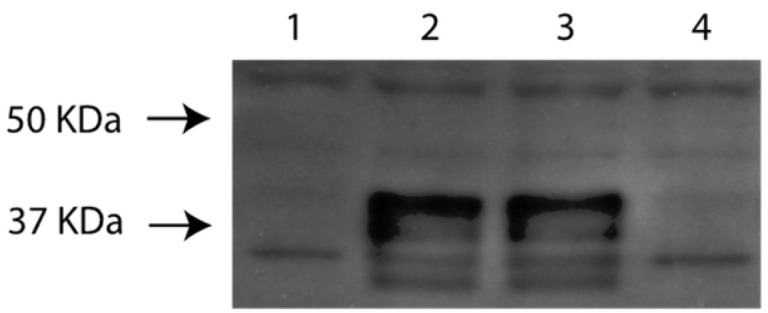
Western blotting analysis of the generated recombinant viruses using guinea pig anti-US9 serum detects US9 polypeptides with relative molecular masses of between 35 and 42 kDa. Markers (lane M) were included to assess the sizes of the US9 polypeptides. Lane 1: RK 13 Mock; Lane 2: Ab4p attB; Lane 3: Ab4pΔORF76R; Lane 4: Ab4pΔORF76; M: molecular weight marker.

The EHV-1 US9-specific antibody reacted with several bands with approximate molecular masses of 35 to 42 kDa in (Figure 4) on Western blots. The EHV-1 Us9-specific bands were absent in mock-infected cells and cells infected with Ab4pΔORF76. 

### 3.5. Pathogenicity of ORF76 Deletion Mutant Virus in Mice

**Figure 5:**
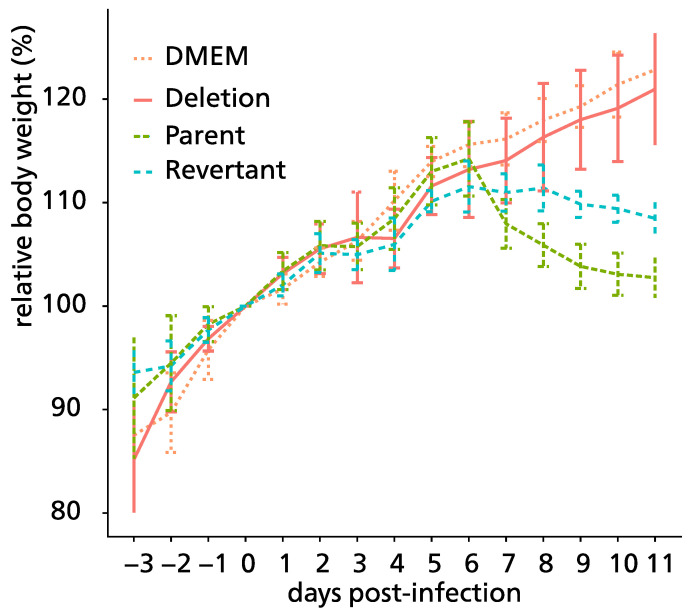
Mean body weight curves of control mice and those inoculated with Ab4p attB (Parent), Ab4pΔORF76 (Deletion), and Ab4pΔORF76R (Revertant) viruses. CBA/N1 mice were infected intranasally with 1 × 10^5^ PFU of the indicated viruses. Body weights were measured from 3 days before inoculation to 11 days post-inoculation. Each data point represents the mean relative body weight for the indicated group. Body weight of day 0 was 100%. Error bars indicate standard errors.

Ab4pΔORF76 inoculated mice were apparently healthy, sound, and gained body weight with no nervous manifestations throughout the experiment period (Figure 5). Nervous manifestations, including arching back, hyperactivity, and paralysis, were observed in mice inoculated with Ab4p attB starting from the fifth day post-infection and Ab4pΔORF76R starting from the sixth day post-infection. 

**Figure 6:**
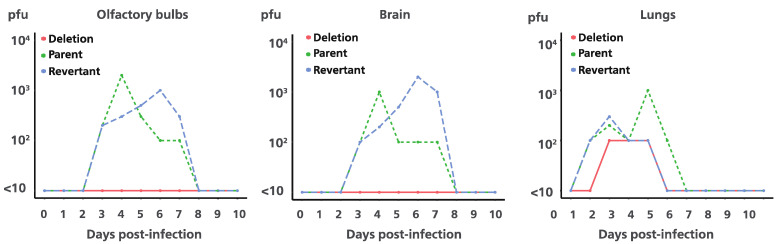
Virus titration in mice organs inoculated with viruses. Ab4p attB (Parent), Ab4p ΔORF76 (Deletion), and Ab4p ΔORF76R (Revertant) were intranasally inoculated to CBA/N1 mice. Olfactory bulbs, brain, and lungs were collected from 0 to 10 days post-infection. Virus titers are expressed as pfu per gram of organ. Detection limit was indicated as <10 pfu per gram of organ.

Viruses were recovered from the infected mice lungs, 1 to 5 dpi for Ab4p attB, 1 to 4 dpi for Ab4p∆ORF76R, and 1 to 3 dpi for Ab4p∆ORF76. Ab4p attB and Ab4p∆ORF76R viruses could be recovered from olfactory bulbs and the brain of infected mice from 3 to 7 dpi, while the Ab4p∆ORF76 virus was not recovered from olfactory bulbs and the brain at all (Figure 6).

**Figure 7:**
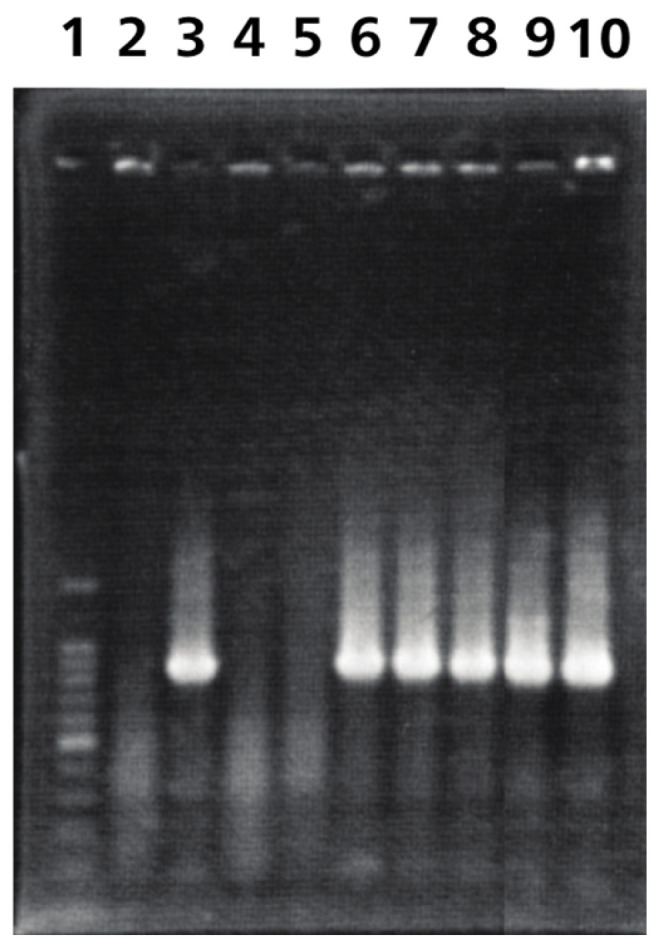
Representative virus DNA detection by PCR. Virus DNA was detected by PCR in DNA extracted from brains of CBA/N1 mice inoculated with viruses. Lane 1: 100 bp marker; Lane 2: negative control (mouse brain DNA without inoculation); Lane 3: positive control (virus DNA extracted from Ab4p attB); Lanes 4, 5: mouse brain DNA inoculated with Ab4p ΔORF76; Lanes 6, 7: mouse brain DNA inoculated with Ab4p attB; Lanes 8, 9, 10: mouse brain DNA inoculated with Ab4p ΔORF76R.

Ab4p attB and Ab4pΔORF76R viruses DNA were detected in olfactory bulbs, brains, and lungs of inoculated mice from 1 dpi. While Ab4p∆ORF76 DNA was not detected at all in olfactory bulbs and brains of mice inoculated with Ab4pΔORF76 (Table 2, Figure 7).

**Figure 8:**
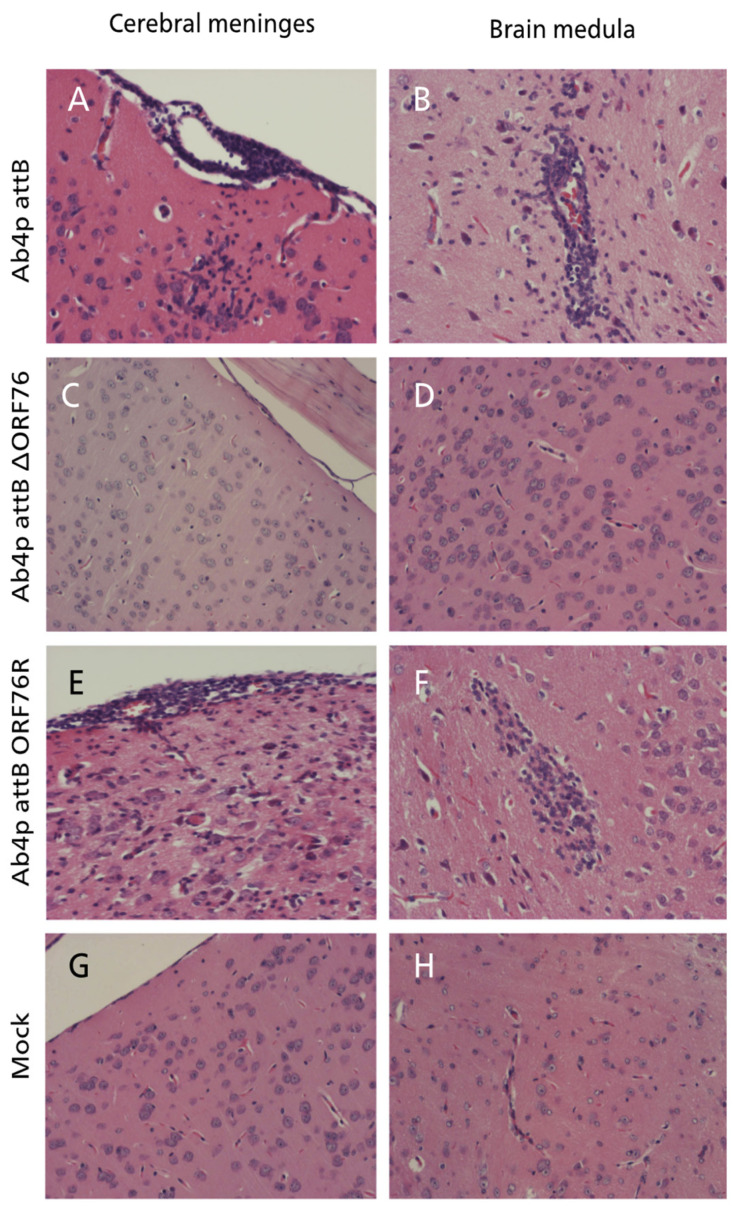
Brain of mice inoculated with Ab4p attB on 5 dpi, showing meningitis in the form of mononuclear cell infiltration and congestion together with gliosis and neuronal necrosis at the cerebral cortex (**A**) and perivascular cuffing with mononuclear inflammatory cells and neuronal necrosis (**B**). Mouse brain inoculated with Ab4p∆ORF76 on 6 dpi, showing normal meninges (**C**) and cerebral cortex (**D**). Mouse brain inoculated with Ab4p∆ on 6 dpi, showing meningitis in the form of mononuclear cell infiltration and congestion together with neuronal necrosis and congestion at the cerebral cortex (**E**) and gliosis and neuronal necrosis (**F**). Mouse brain with mock virus infection on 7 dpi, showing normal meninges (**G**) and brain (**H**). Hematoxylin and eosin staining, ×200.

At necropsy, no gross abnormalities were observed in any of the inoculated mice. Histopathological examination of mice infected with each virus revealed mild rhinitis, multiple foci of necrosis of the olfactory epithelial cells, along with inflammatory cells infiltrates within the mucosa admixed with the desquamated epithelial cells in the nasal cavity. The olfactory bulbs of mice inoculated with Ab4p attB or Ab4p∆ORF76R showed typical encephalitis at 4 and 6 days post-infection, respectively, while no pathological changes were recorded in mice inoculated with Ab4pΔORF76. The brains of mice infected with Ab4pΔORF76 showed no significant pathological changes, while the brains of Ab4p attB and Ab4p∆ORF76R infected groups showed lymphocytic meningoencephalitis, consisting of neuronal degeneration and necrosis, perivascular aggregates of mononuclear cells and varying degrees of focal or diffuse gliosis (Figure 8). No abnormalities were found in other organs except for interstitial pneumonia in all virus-infected groups.

**Figure 9:**
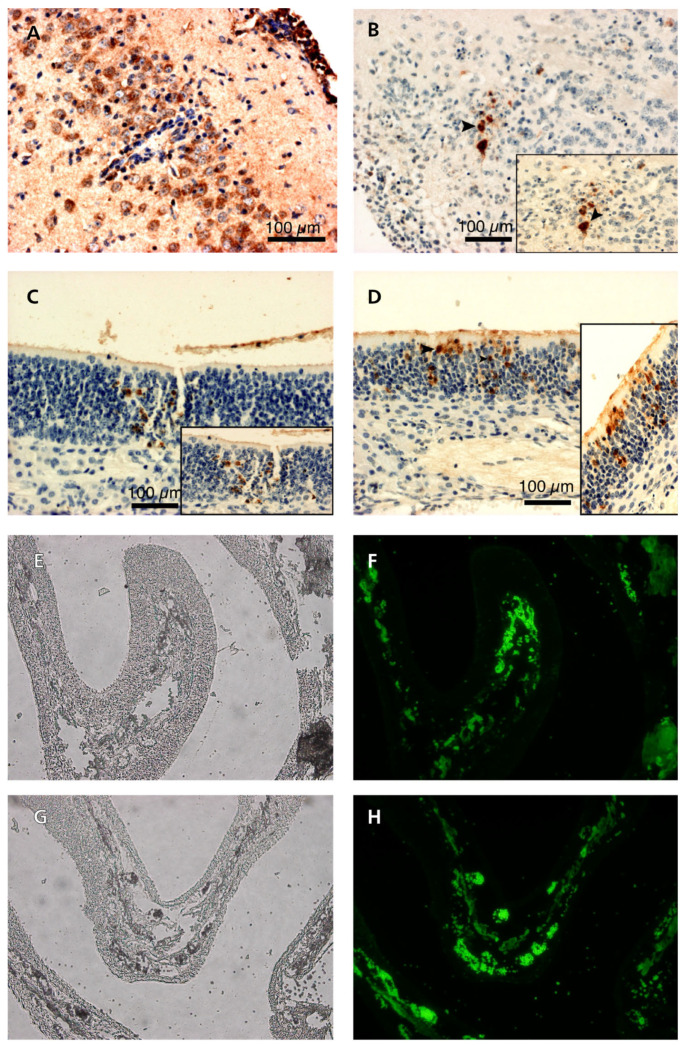
Immunoperoxidase of sections of brains and olfactory epithelium of mice infected with Ab4p attB (**A**,**C**) and Ab4p∆ORF76R (**B**,**D**). The primary antibody was anti-EHV-1 rabbit serum, followed by biotinylated anti-rabbit IgG antibody. Immunofluorescence of sections of olfactory epithelium of mice infected with Ab4p attB (**F**) and Ab4p∆ORF76 (**H**). Magnification (**A**–**D**) ×400. Bar, 100 µm. Visible light images (**E**,**G**) and fluorescence images (**F**,**H**). Magnification (**E**–**H**) ×40. The primary antibody was anti-US9 guinea pig serum, followed by FITC-labeled anti-guinea pig IgG antibody.

EHV-1 antigens were detected by immunohistochemical and immunofluorescence reactions in the cytoplasm of degenerating olfactory epithelial cells in the nasal cavity of mice infected with each of Ab4p attB, Ab4p∆ORF76R, and Ab4pΔORF76. EHV-1 antigen was detected in the brains and olfactory bulbs of mice inoculated with Ab4p attB or Ab4p∆ORF76R (Figure 9) but not in those of mice infected with Ab4p∆ORF76.

## 4. Discussion

The US9 gene is conserved in most of the alphaherpesviruses. US9 is not essential for replication in cell culture and plays no role in the cell-to-cell spread of HSV-1 [27,45], PRV [46], and BHV-5 [38]. Our results show that EHV-1 ORF76 (US9) is also not involved in viral replication in cell cultures (RK13, MDBK, and FHK) or cultivated primary mouse neuronal cells and has no role in cell-to-cell spread. The histopathological and immunohistochemical results of this study prove that US9 has an important role in EHV-1 anterograde neuronal transport in the olfactory pathway and so EHV-1 neuropathogenesis. 

The EHV-1 US9 gene is 660 nucleotides and encodes a protein of 219 amino acids and 22.287 kDa. Using the Western blot assay, US9 polyclonal antibodies reacted with several polypeptides between 35 and 42 kDa. The EHV-1 US9-specific bands were absent in mock-infected cells and cells infected with the EHV-1 US9 deleted mutant. The molecular masses of the EHV-1 US9-specific bands were higher than expected, which suggests that the EHV-1 US9 protein had undergone posttranslational modifications. The US9 ORFs of HSV-1, PRV, BHV-5, and BHV-1 encode proteins with predicted molecular weights of 10.0, 10.8, 13.7, and 14.7 kDa, respectively. However, US9-specific polyclonal antibodies precipitated several proteins with molecular weight ranges of 12–25 kDa in HSV-1, 17–20 kDa in PRV, 15–20 kDa in BHV-5 and 28–32 kDa in BHV-1. In each of these viruses, PRV, HSV-1, BHV-5, and BHV-1, US9 is phosphorylated [38,47,48]. It is believed that phosphorylation alters the charge of the SDS coating of the protein, which slows protein migration in the gel, so the protein seems to have a higher, apparent molecular weight [49]. Our finding that the EHV-1 US9 has multiple bands of apparent higher molecular mass than expected supports the notion that the alphaherpesvirus Us9 proteins are phosphorylated and suggests that phosphorylation of Us9 has a functional role.

The role of the EHV-1 ORF76 (US9) gene in vivo was evaluated in a CBA/N1 mouse infection model. In mice intranasally inoculated with Ab4p attB and Ab4pΔORF76R, the viruses spread in the olfactory pathway and caused histopathological lesions similar to those previously described in wild-type EHV-1 intranasally inoculated mice [23,25,43]. In contrast, in mice intranasally inoculated with the EHV-1 US9 deletion mutant (Ab4pΔORF76), the virus failed to invade the CNS as indicated by several lines of evidence, including absence of nervous manifestations, normal body weight gain, no mortalities, normal histopathological finding of the olfactory bulbs and brain, and no virus antigen detection within the CNS by viral isolation, PCR or immunostaining. These results indicate that the ORF76 (US9) plays an essential role in the anterograde spread of the EHV-1 virus.

In mice intranasally inoculated with BHV-1, BHV-5 [37,50], and EHV-9 [51,52], after initial replication in the olfactory mucosa, the viruses are transported to the olfactory bulbs, and then to deeper tissues of the brain through olfactory tract neurons. In the nasal mucosa, the Ab4pΔORF76 replicated efficiently, just like Ab4p attB and Ab4pΔORF76R viruses. Immunostaining detected Ab4pΔORF76 antigen in the olfactory epithelium receptor neurons but not in the bulbs, indicating that Ab4pΔORF76 was not transported to the olfactory bulbs. Our result supports numerous other reports that have shown that Us9 has a role in the anterograde spread of alphaherpesviruses [38,46,53,54,55]. Roles of US9 in vivo might be conserved among alphaherpesviruses.

## 5. Conclusions

The gene product of EHV-1 ORF76 (US9) is not essential for the replication of EHV-1 in cell culture or cultivated neurons and is not involved in EHV-1 cell-to-cell spread, but it plays an important role in the anterograde spread of EHV-1.

## Figures and Tables

**Table 1:** Sequences of primers used for the construction and analysis of generated mutant viruses.

Target	Primer	Sequence
rpsL-neo cassette	1	5′-TTT CCC TCT CAG CGA TCA CTT TTC ACC ACC GAA GAA CAG GCC CTC ATC G**GG GCC TGG TGA TGA TGG CGG GAT CG**-3′
	2	5′-GGG CTG TTG TGG GGT AAA AGG TGG TGT TAC GGA AAC ACG CGT GCC AAG **AAT CAG AAG AAC TCG TCA AGA AGG CG**-3′
rpsL-ORF76	3	5′-TTT CCC TCT CAG CGA TCA CTT TTC ACC ACC GAA GAA CAG GCC CTC ATC GG-3′
	4	5′-GGG CTG TTG TGG GGT AAA AGG TGG TGT TAC GGA AAC ACG CGT GCC AAG AA-3′
ORF76 with EcoRI and NotI	5	5′-ccg gaa ttc ATG GAG AAG GCG GAG GCT GCC GCA-3′
	6	5′-aag gaa aaa agc ggc cgc TTA CGG AAA CAC GCG TGC CAA GAA-3′
ORF76	7	5′-CTA CCG TGG AAG CGG TAT GT-3′
	8	5′-ATT CTC AGA AGC AGC GGT GT-3′
ORF75	9	5′-CAA CCC TGT CAG AAA CAG CA-3′
	10	5′-GGG GGA GGT AGA GTT TCC AG-3′
ORF67	11	5′-TCG GCC CTT ATG TAA TAG CG-3′
	12	5′-CTC CTA CTT CAG GCG GTG TC-3′
ORF30	13	5′-gtc agg ccc aca aac ttg at-3′
	14	5′-act cgg ttt acg gat tca cg-3′

Sequences in bold uppercase represent the rpsLneo sequence. Sequences in lowercase with underlining are restriction sites for EcoRI and NotI.

**Table 2:** Virus DNA detection in mice organs inoculated with Ab4p attB, Ab4p ΔORF76, and Ab4p ΔORF76R.

Virus	Organ ^1^	Virus DNA Detection on Days Post-Infection										
		0	1	2	3	4	5	6	7	8	9	10
Ab4p attB	O	-	+	+	+	+	+	+	+	+	+	+
	B	-	+	+	+	+	+	+	+	+	+	+
	L	-	+	+	+	+	+	+	+	+	+	+
Ab4p ΔORF76	O	-	-	-	-	-	-	-	-	-	-	-
	B	-	-	-	-	-	-	-	-	-	-	-
	L	-	-	+	+	+	+	+	+	+	-	-
Ab4p ΔORF76R	O	-	+	+	+	+	+	+	+	+	+	+
	B	-	+	+	+	+	+	+	+	+	+	+
	L	-	+	+	+	+	+	+	+	+	+	+

^1^ O, olfactory lobe; B, brain; L, lung. +, Virus DNA was detected; -, Virus DNA was not detected.

## Data Availability

Data are contained within the article.
